# Evaluation de la tolérance et de l'efficacité du traitement de l'hépatite virale chronique C chez les patients drépanocytaires homozygotes

**DOI:** 10.11604/pamj.2015.20.99.6003

**Published:** 2015-02-04

**Authors:** Mohamed Ould Mohamed El Agheb, Jean-Didier Grange

**Affiliations:** 1 Service d'Hépato Gastro Enterologie Hôpital Tenon, 4 Rue de La Chine, 75020 Paris

**Keywords:** Hémoglobinopathies, anemié hémolytique congénitale, traitement combiné, réponse virale soutenue, hemoglobinopathies, congenital hemolytic anemia, traitement combine, sustained viral response

## Abstract

La drépanocytose est une maladie génétique à transmission autosomale codominante. Les patients homozygotes ont des crises hémolytiques qui génèrent les symptômes cliniques. Il s'agit d'une maladie fréquente. En France, la plupart des cas hospitalisés sont observés en Ile de France et aux Antilles. Le pronostic des patients s'est beaucoup amélioré ces dernières années. L’âge médian au décès a doublé en 20 ans. Il est passé de 18 à 36 ans, témoignant d'une meilleure prise en charge des malades. De ce fait, les complications hépatiques de la maladie, et notamment celles liées à l'hépatite virale chronique C, sont de plus en plus fréquentes. La Ribavirine étant contre indiquée dans le traitement de l'hépatite C chez les malades atteints d'anémie hémolytique (talassémie, drépanocytose), il n'existe que très peu de cas publiés dans les littératures et aucun cas traité par trithérapie antivirale incluant un inhibiteur de protéase. Le but de ce travail était d’évaluer la tolérance, l'efficacité du traitement de l'hépatite virale chronique C chez des patients drépanocytaires homozygotes. Dans la cohorte de patients drépanocytaires homozygotes adultes de l'hôpital Tenon (n = 560), la prévalence de l'hépatite C chez les derepanocytaires était de 7% (n = 38) en 2012. Il s'agissait de 15 hommes et 23 femmes âgés de 20 à 59 ans. Vingt-cinq patients avaient reçu plus de 10 transfusions et 13 patients avaient reçu moins de 10 transfusions au cours des années précédant le bilan. La répartition des génotypes était: G1 (n = 7); G2 (n = 2); G3 (n = 1); G4 (n = 6); G inconnu (n = 22). Neuf patients ont été traités dont 8 par bithérapie (Peg/Rbv) et 1 par trithérapie (télaprevir). La posologie de ribavirine était supérieure ou égale à 800mg/jour chez 7 patients et inférieure à cette dose chez les deux autres patients. Le score METAVIR de fibrose était: F1 (n = 3), F2 (n = 4) et F 3 (n = 1);un patient n'a pas pu etre biopsié. Aucun patient n'a présenté de crise vaso-occlusive. Un seul patient a du être transfusé. Chez les 8 autres patients, la tolérance hématologique était excellente; le taux d'hémoglobine moyen était supérieur au taux moyen pré-thérapeutique après 1 et 3 mois de traitement. Une réponse virologique complète était observée chez tous les patients en fin de traitement et une réponse virale soutenue (SVR 24) était obtenue chez 6 patients. La cohorte des patients atteints de drépanocytose de l'Hôpital Tenon est une des plus importantes décrites dans la littérature. La prévalence de l'infection par le virus C est de 7% chez les patients homozygotes SS faisant des crises vaso occlusive. Cette série confirme que la prévalence dépend du nombre de transfusions (supérieures ou inférieures à 10 unités d’érythrocytes) avant le diagnostic. Il confirme également la bonne tolérance de la prise de ribavirine chez ces patients ayant une anémie hémolytique congénitale. Aucune crise vaso-occlusive n'a été observée. Le nombre de transfusion n'est pas augmenté. La bonne tolérance également observée chez le malade traité par trithérapie peut ouvrir la voie à l’évaluation des nouveaux antiviraux directs. Un suivi régulier conjoint par un hépatologue et un hématologue est impératif dans cette population fragile.

## Introduction

La drépanocytose est une maladie génétique à transmission autosomale codominante. Les patients homozygotes ont des crises hémolytiques qui génèrent les symptômes cliniques. Il s'agit d'une maladie fréquente. En France, la plupart des cas hospitalisés sont observés en Ile de France et aux Antilles [1]. Le pronostic des patients s'est beaucoup amélioré ces dernières années. La médiane de l’âge du décès est passée de 18 à 36 ans en 20 ans [1]. Actuellement l'espérance de vie des malades à la naissance est de 45 ans, 95% des patients atteignant l’âge adulte. Chez les enfants, en Ile de France, les complications infectieuses, les crises de séquestration splénique et les accidents vasculaires cérébraux sont les principales causes de décès. Chez l'adulte, avec l'amélioration globale du pronostic, des complications hépatologiques, notamment celles liées à l'hépatite virale chronique C, deviennent de plus en plus importantes. Malheureusement, les mentions légales et la Ribavirine stipulent que son emploi est contre indiqué chez les malades atteints d'hémoglobinopathies telles que les talassémies et la drépanocytose. De ce fait, il n'existe aucune grande série de malades traités dans les littératures. Dans une étude préliminaire effectuée dans le service d'hépato-gastroentérologie de l'Hôpital Tenon, un traitement par Interféron Pégylé et Ribavirine avait été effectué chez 6 patients atteints de drépanocytose homozygote [2]. Au plan hématologique, la tolérance avait été bonne, suggérant que la dose initiale de Ribavirine pourrait être administrée d'emblée selon les mentions légales de ce produit. Actuellement tous les schémas thérapeutiques incluent l'utilisation d'Interféron Pégylé et de Ribavirine. Des travaux ont montré qu'il était important pour le succès du traitement d'administrer la dose optimale de Ribavirine. Chez les patients talassémiques, une méta-analyse a montré que les patients traités par Interféron Pégylé et Ribavirine avaient une augmentation de leurs besoins transfusionnels de 30 à 40%, sans modification de la fréquence des autres effets indésirables. Chez les patients talassémiques, nous avons pu contribuer à l’élaboration de recommandation internationale publiée le 15 juin 2010 [3]. Les publications actuelles chez les malades atteints de drépanocytose sont pour le moment insuffisantes pour établir des recommandations. Le but de ce travail était d’évaluer la tolérance et l'efficacité du traitement de l'hépatite virale chronique C chez les patients drépanocytaires homozygotes.

## Méthodes

### Patients

En 2012, la file active de patients drépanocytaires du service d'Hématologie de l'Hôpital Tenon comportait 785 patients. La drépanocytose était diagnostiquée pour chaque patient sur les données de l’électrophorèse de l'hémoglobine, de l'enquête familiale et des tests génétiques. Cinq cent soixante patients étaient homozygotes SS dans cette cohorte, parmi lesquels 38 avaient une sérologie virale C positive. La virémie C était positive chez 16 patients. Ces 38 patients (âge moyen: 40±20 ans; 15 hommes-23 femmes) suivis de 1993 à 2013 ont été inclus dans notre étude. Neuf patients ont été traités pour leur infection virale C. Les critères de traitement étaient une augmentation persistante des ALAT, une positivité de l'ARN du VHC persistant plus de 6 mois. Les données épidémiologiques de la cohorte de patients sont decrites dans le Tableau 1.

**Tableau 1:** Données épidémiologiques de la cohorte de patients atteints de drépanocytose de Tenon

	N
Age moyen au moment du bilan	40 + /-20 ans
Sexe (H/F)	15H/23F
Patients homozygotes SS	560
Patients Acs anti HCV positifs	38
Patients ARN VHC positif	16

### Méthodes

Avant le début de leur traitement, les patients avaient une évaluation de l'infection virale C et de la fonction hépatique comprenant: un dosage de la charge virale C, une détermination du génotype viral, une détermination de la fibrose hépatique soit par biopsie hépatique soit par une méthode indirecte (Fibrotest ou Fibroscan), un dosage des paramètres biologiques hépatiques (ASAT, ALAT, PAL, GGT, TP) et de l'hémoglobinémie. Toutes les biopsies étaient côtés selon le score de METAVIR pour l'activité nécrotico inflammatoire (4 stades de 0 à 3) et la fibrose (5 stades 0 à 4). Le traitement antiviral C associait un interféron pégylé à de la ribavirine. L'interféron était donné d'emblée à la dose optimale (1,5µg/kg ou 180 µg) en une injection hebdomadaire. Compte tenu des risques d'anémie hémolytique, la ribavirine était introduite à la dose de 400 à 600 mg en deux prises quotidiennes, puis augmentée progressivement en fonction des contrôles hématologiques jusqu’à la dose optimale. La durée prévue de traitement était de 24 semaines pour les génotypes 2 et 3 et de 48 semaines pour le génotype 1 et 4. Un patient de génotype 1b, a été traité par une trithérapie associant telaprevir, ribavirine et interféron pegylé. Les patients ont été surveillés tous mois durant le traitement et à 6 mois après la fin du traitement. A chaque visite étaient évalués: l'observance du traitement, l'adaptation de la posologie, les effets secondaires cliniques, les besoins transfusionnels depuis la dernière consultation,la numération formule sanguine,le bilan hépatique et la charge virale. La réponse virale était définie comme la négativation de la recherche de l'ARN viral C par PCR. Elle était évaluée mensuellement pendant la durée du traitement, en fin de traitement et 6 mois après la fin du traitement. Les patients étaient classés comme: non répondeur en cas d'absence de négativation de la virémie, de répondeur en cas de négativation de la virémie, de répondeur-rechuteur en cas de nouvelle positivité de la virémie après un résultat négatif. La réponse virale soutenue(RVS), a été définie comme la persistance de la réponse virale 6 mois après la fin du traitement. Statistiques: Les résultats sont exprimés en sommes et médianes pour les données qualitatives. La moyenne ± ESM a été calculée pour les variables quantitatives. La prévalence de l'infection par le VHC dans la cohorte est exprimée en pourcentage par rapport à l'ensemble des malades homozygotes SS. Les statistiques ont été réalisées avec un Tableur Excel(Microsoft). Les données epidemiologiques chez les patients infectés par le virus de l′hepatite C sont reprises dans leTableau 2.

**Tableau 2:** Données épidémiologiques chez les patients drépanocytaires infectés par le virus C à Tenon

N	38
Génotype (1/2/3/4/ nd)	7/2/1/6/22
Nombre de transfusions avant traitement	
> 10	25
< 10	13
Score de fibrose par élastométrie (kPa; extrèmes)	10 + /-4

## Résultats

**Figure 1:**
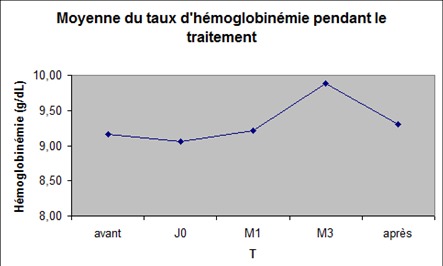
Variation des concentrations moyennes d'hémoglobine au cours du traitement chez les huit patients traités

Neuf des trente-huit patients avec une sérologie C positive ont été traités par bi ou tri thérapie antivirale. Les caractéristiques démographiques et virales de ces patients sont résumées dans les (Tableau 3, Tableau 4). Le nombre total de transfusions reçues par les patients avant l'evaluation de la prise en charge était supérieur à 10 chez 25 patients (65%) et inférieur à 10 chez 13 patients (35%). L’âge médian des patients infectés par le virus C au moment de l’évaluation était de (40 + _20 ans). La réponse virale en fin de traitement, associée à la normalisation des ALAT était observée chez les 9 patients. Une réponse virale soutenue était observée chez 6 patients (67%). Sept patients sur 9 (78%) ont reçu et toléré une dose de ribavirine de 800 + /-200 mg/j. Avant le traitement, seul un patient avait une hémoglobinémie fluctuant entre 5 et 8 g/dL. Pendant la durée de traitement, l'hémoglobinémie était supérieure au seuil de transfusion chez tous les malades à M2, chez 8/9 entre M3 et M4 et à nouveau chez tous les malades de M5 jusqu’à l’évaluation finale à M6 post traitement. Les moyennes de l'hemoglobine pendant la periode d'observation sont donées dans la Figure 1.

**Tableau 3:** Données épidémiologiques et virales des patients traités par bi ou tri thérapie

N	8
Bithérapie/trithérapie	7/1
Génotype viral (1/2/3/4/nd)	4/2/1/2/0
Charge virale moyenne (extrêmes)	1640000 + /-500000ui/ml
Fibroscan kPa (extrêmes)	7 + /-3
Score METAVIR activité (1/2)	7/1
Score METAVIR fibrose (1/2/3/4)	3/4/1/0
Traitement par Hydréa	3
Posologie initiale de ribavirine (400/600/800/1000)	2/3/3/1
Posologie finale de ribavirine (600/800/1000)	2/6/1
Dose cumulée moyenne de ribavirine	800 + /-200mg/jr

**Tableau 4:** Patients ayant été traités par bitherapie

Patient	Age	Genotype	metavir	FibroscanKPa	Charge virale	traitement	rbv initiale	rbv finale	durée	Reponse en fin traitement	RVS
Patient 1	1972	4	A1F2	7.9	0.2méq	Peg/Rbv	400 mg	800mg	48 S	OUI	OUI
Patient 2	1976	1a +4	A2F2	ND	0.3méq	Peg/Rbv	400mg	1000mg	48s	OUI	OUI
Patient 3	1973	2	A1F2	ND	1200000ui/ml	Peg/Rbv	600MG	600MG	24S	OUI	NON
Patient 4	1982	4	A1F2	9.9	100331 UI/ML	Peg/Rbv	800MG	800MG	36S	OUI	OUI
Patient 5	1965	1a	A1F3	10.2	6126378 UI/ML	Peg/Rbv	1000MG	800MG	48S	NON	NON
Patient 6	1969	2	A1F1	5.6	6360000UI/ML	Peg/Rbv	800MG	800MG	24	OUI	OUI
Patient 7	1980	3	A1F1	4.4	259000UI/ML	Peg/Rbv	600MG	600MG	18S	OUI	OUI
Patient 8	1986	1b	A1F1	7.6	49000UI/ML	Peg/Rbv	600mg	800mg	48	OUI	NON
Patient 9	1974	1b	NF	6,6	668000 UI/ml	Peg/Rbv/telaprevir	800MG	800MG	48	OUI(2)	OUI

## Discussion

Les patients drépanocytaires homozygotes SS sont exposés à une infection au virus de l'hépatite c à cause des transfusions multiples. Notre cohorte de patients drépanocytaires est une des plus importantes. La prévalence de l'HVC chez les patients drépanocytaires est de7%. Cette prevalence dépend du nombre de transfusions reçues,elle varie de 65% chez patients infectés ayant reçu plus de 10 transfusions à 35% chez ceux ayant reçu moins de 10 transfusions. Ces résultats sont comparables à ceux de la littérature. Dans une cohorte de 99 patients [4], la prévalence globale de l'HCV chez des patients drépanocytaires était de 10% [4]. Elle variait de 23,3% chez les patients ayant reçu plus de 10 unités globulaires à 8% chez ceux ayant reçu moins de 10 unités globulaires. Devaut et al [5] chez 121 patients avec drépanocytose, trouvaient une prévalence de 20,7%. Ils constataient également une variation de cette prévalence en fonction du nombre de transfusions reçues, 30% si plus de 10 unités globulaires transfusées, 8,6% si moins de 10 unités globulaires transfusées [5]. Peu de patients drépanocytaires infectés par le VHC ont été traités par une bithérapie et aucun par trithérapie. Dans notre série 8 des patients ont été traités dont 7 par bitherapie et un par trithérapie. Un patient programmé pour trithérapie n'a pas été inclus dans l’étude car il a développé une crise vaso-occlusive sévère qui a fait différé le projet de traitement anitiviral. 29 patients (76%) n'ont pas été traités. Dans 22(57%) cas la PCR du VHC était négative, témoignant d'une guérison spontanée de la maladie virale. Ce chiffre est plus important que celui habituellement observé dans la population générale des patients traités pour hépatite C chronique 15 à 20% [6]. Cette difference sur la prevalence peut s'expliquer par la qualité des mesures de surveillance transusionnelle selon les lieux d’étude.

Le taux de RVS observé dans notre série est comparable à celui obtenu dans la population générale et dans les courtes séries publiées chez les patients drépanocytaires. Ayyub et al [6] ont traité de 2003 à 2006, huit patients drépanocytaires infectés par VHC par peg-interferon et ribavirine pendant 48S. Les 8 patients avaient une réponse virologique en fin de traitement et 5 une réponse virale soutenue [6]. Il n'ya pas eu de modification significative du taux d'hémoglobine au cours des 12mois de traitement, aucune transfusion ni crise vaso occlusive n'a été signalé en cours de traitement. Ancel et al [2] ont rapportés une série de 11 patients dont 6 drépanocytaires et 5 avec beta thalassémies majeure infectés par le VHC traités par interferonpegylé et ribavirine. Cinq ont eu une RVS,32.5% des thalassémiques ont eu besoin d'une transfusion sanguine alors qu'aucun drépanocytaire n'a eu besoin de transfusion. Une série plus importante doit néanmoins confirmer ce résultat. Aucun patient dans la littérature n'a été traité par une trithérapie. Bien que s'agissant d'un cas unique, notre patient traité par télaprevir, peginterféron et ribavirine, n'a pas eu d'effet indésirable important. Ce résultat doit être confirmé sur une série plus importante. Il permet cependant d'envisager le traitement des patients

## Conclusion

La cohorte des patients atteints de drépanocytose de l'Hôpital Tenon est une des plus importantes décrites dans la littérature. La prévalence de l'infection par le virus C est de 7% chez les patients homozygotes SS faisant des crises vaso occlusive. Cette série confirme que la prévalence dépend du nombre de transfusions (supérieures ou inférieures à 10 unités d’érythrocytes) avant le diagnostic. Il confirme egalement les données de la littérature qui montrent une bonne tolérance de la prise de ribavirine chez ces patients ayant une anémie hémolytique congénitale. Aucune crise vaso-occlusive n'a été observée. Le nombre de transfusion n'est pas augmenté. La bonne tolérance également observée chez le malade traité par trithérapie peut ouvrir la voie à l’évaluation des nouveaux antiviraux directs. Un suivi régulier conjoint par un hépatologue et un hématologue est impératif dans cette population fragile.
